# Dosimetric comparison study between intensity modulated radiation therapy and three‐dimensional conformal proton therapy for pelvic bone marrow sparing in the treatment of cervical cancer

**DOI:** 10.1120/jacmp.v11i4.3255

**Published:** 2010-08-15

**Authors:** William Y. Song, Soon N. Huh, Yun Liang, Greg White, R. Charles Nichols, W. Tyler Watkins, Arno J. Mundt, Loren K. Mell

**Affiliations:** ^1^ Department of Radiation Oncology University of California San Diego La Jolla CA; ^2^ Department of Radiation Oncology University of Florida Proton Therapy Institute Jacksonville FL USA

**Keywords:** cervical cancer, proton therapy, IMRT, pelvic bone marrow, DVH analysis

## Abstract

The objective was to compare intensity‐modulated radiation therapy (IMRT) with 3D conformal proton therapy (3DCPT) in the treatment of cervical cancer. In particular, each technique's ability to spare pelvic bone marrow (PBM) was of primary interest in this study. A total of six cervical cancer patients (3 postoperative and 3 intact) were planned and analyzed. All plans had uniform 1.0 cm CTV‐PTV margin and satisfied the 95% PTV with 100% isodose (prescription dose=45Gy) coverage. Dose‐volume histograms (DVH) were analyzed for comparison. The overall PTV and PBM volumes were 1035.9±192.2 cc and 1151.4±198.3 cc, respectively. In terms of PTV dose conformity index (DCI) and dose homogeneity index (DHI), 3DCPT was slightly superior to IMRT with 1.00±0.001,1.01±0.02, and 1.10±0.02,1.13±0.01, respectively. In addition, 3DCPT demonstrated superiority in reducing lower doses (i.e., V30 or less) to PBM, small bowel and bladder. Particularly in PBM, average V10 and V20 reductions of 10.8% and 7.4%(p=0.001 and 0.04), respectively, were observed. However, in the higher dose range, IMRT provided better sparing (>V30). For example, in small bowel and PBM, average reductions in V45 of 4.9% and 10.0%(p=0.048 and 0.008), respectively, were observed. Due to its physical characteristics such as low entrance dose, spread‐out Bragg peak and finite particle range of protons, 3DCPT illustrated superior target coverage uniformity and sparing of the lower doses in PBM and other organs. Further studies are, however, needed to fully exploit the benefits of protons for general use in cervical cancer.

PACS number: 87.55.D‐, 87.55.dk

## I. INTRODUCTION

In the last decade, concurrent chemotherapy with whole‐pelvic external beam radiotherapy has emerged as the primary treatment option for locally advanced cervical cancer.^(^ ^1^ ^–^ ^5^ ^)^ Although shown to increase tumor control and overall survival, the use of concurrent chemotherapy with radiation also increases the acute hematologic toxicity (HT), thus limiting the further utilization of chemotherapy for achieving maximum tumor control.^(^ ^4^ ^–^ ^6^ ^,^ ^7^ ^)^ This is unfortunately due to the fact that most of the total body bone marrow reserve is located within the lower lumbar spine and pelvic bones.^(^ ^7^ ^,^ ^8^ ^)^

In the hopes of reducing related toxicities, many investigators looked into intensity‐modulated radiation therapy (IMRT) as a potential replacement to the traditional two to four field arrangements for minimizing radiation damage to surrounding tissues by sculpting the dose distribution around the target volume.^(^ ^7^ ^–^ ^9^ ^–^ ^16^ ^)^ Dosimetric analyses^(^ ^10^ ^–^ ^11^ ^,^ ^13^ ^‐^ ^14^ ^)^ as well as acute toxicity studies^(^ ^7^ ^,^ ^12^ ^)^ both revealed the advantages of IMRT for target coverage and normal tissue sparing for pelvic treatments. In particular, the advantages of IMRT for bone marrow sparing compared with conventional techniques have been shown.^(^ ^11^ ^,^ ^14^ ^)^

In recent analyses, it was shown that the volume of pelvic bone marrow (PBM) and lumbosacral bone marrow (LSBM) receiving 10 and 20 Gy are significantly associated with acute HT events in patients undergoing concurrent chemotherapy and IMRT.^(^ ^15^ ^–^ ^16^ ^)^ Therefore, search for techniques to further minimize the low doses (in the range of 10–20 Gy) to PBM seems logical. One potential modality that may be able to do this is protons.^(^ ^17^ ^,^ ^18^ ^)^ With its attractive physical characteristics (e.g., low entrance dose, spread‐out Bragg peak, and finite particle range), proton therapy can potentially reduce the dose to PBM more so than IMRT. In fact, a number of investigators looked into the potential use of protons for cervical cancer treatments and concluded that doses to organs at risk (OARs) are reduced.^(^ ^19^ ^–^ ^22^ ^)^ However, none have looked at PBM sparing specifically.

The purpose of this study was to compare IMRT with three‐dimensional conformal proton therapy (3DCPT) for dosimetric benefits in the treatment of cervical cancer. In particular, each technique's ability to spare PBM was of primary interest in this study. To do this, we developed treatment plans using IMRT and proton therapy specifically designed to maximize the bone marrow sparing (BMS) intent. The dose‐volume histograms (DVH) were the main means of comparison.

## II. MATERIALS AND METHODS

### A. Simulation

Six cervical cancer patients (3 postoperative and 3 intact) treated at the University of California, San Diego (UCSD) were selected for analysis. All patients were immobilized with customized Vac Loc bags encompassing the upper and lower body before a CT scan of the pelvic region was performed (GE wide‐bore 4‐slice LightSpeed CT scanner, GE Healthcare, USA). Oral, intravenous and rectal contrasts were administered in select patients to aid in the delineation of normal and target tissues.

### B. Volume definition

Following ICRU 50 and 78 recommendations,^(^ ^23^ ^,^ ^24^ ^)^ the clinical target volume (CTV) and OAR structures were contoured on individual simulation CT studies. Detailed description of the delineations can be found in previous publications.^(^ ^10^ ^,^ ^12^ ^)^ In brief, the CTV consisted of the pelvic and presacral lymph nodes, uterus and cervix (if present), upper vagina, and parametrial tissues. OARs included bowel, rectum, bladder and PBM (including lumbosacral spine, os coxae, and proximal femora). The overall PTV and PBM volumes were $1035.9\pm 192.2\;{\text{cm}}^{3}$ and $1151.4\pm 198.3\;{\text{cm}}^{3}$, respectively. All plans had uniform 1.0 cm CTV‐PTV margin and were normalized to cover the 95% PTV with 100% isodose, with the prescription dose of 45 Gy in 1.8 Gy per fraction.

### C. IMRT planning

**Figure 1:**
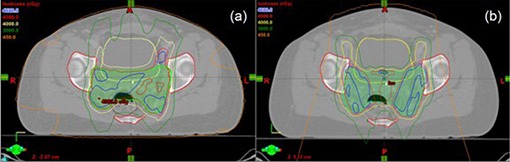
Axial computed tomography image with isodose distribution from (a) intensity modulated radiation therapy plan, and (b) 3D conformal proton therapy plan. Highlighted in green is the planning target volume.

For IMRT, all of the treatment planning was performed by an experienced dosimetrist at UCSD. For each patient, 6 MV, eight‐field, non‐equidistant, coplanar, sliding‐window IMRT technique was planned on the Eclipse treatment planning system (TPS) (Varian Medical Systems, Palo Alto, CA). The beam placement and dose‐volume constraints were selected, monitored and interactively adjusted to best spare the PBM while maintaining adequate target coverage. Table 1 lists the planning goals used for general guidance of both IMRT and proton therapy planning. Figure 1(a) illustrates the typical dose distribution achieved by this IMRT technique.

**Table 1:** General planning guideline used for both intensity‐modulated radiation therapy and 3D conformal proton therapy plan design.

*Structure*	*Dose‐Volume Criteria*
Planning Target Volume	100% dose to 95% volume; 97% dose to 97% volume; 95% dose to 99% volume
Skin	<1% greater than 120% dose, <5% greater than 115%, <10% greater than 110%
Pelvic Bone Marrow	V5<95%,V10<80%,V20<60%,V30<50%,V40<35%
Bowel	As low as possible
Bladder	As low as possible
Rectum	As low as possible

### D. Proton planning

For 3DCPT, all of the treatment planning was performed by an experienced planning physicist at the University of Florida Proton Therapy Institute (UFPTI). For each patient, two posterior oblique, and one anterior‐inferior oblique (with 90° couch kick) fields were planned on the Eclipse TPS with proton planning module. This three‐field technique was chosen, after evaluating multiple different beam orientations, due to the following three reasons: 1) best minimized the PBM dose while achieving excellent target coverage (that is, lateral fields are detrimental to the PBM sparing, while AP/PA‐type fields are the best), 2) anterior field with couch kick largely misses the bowel and also it is less prone to day‐to‐day variation in its proton beam path length due to the daily abdominal content change, and 3) two posterior oblique fields' axes align with the ilium curvature, thus minimizing dose to this structure and hence the total PBM dose. Figure 1(b) illustrates the typical dose distribution achieved by this 3DCPT technique.

In terms of the proton beam delivery technique, the uniform scanning with aperture and range compensator was used. This technique can deliver a maximum water equivalent thickness (WET) range of 28 cm and 250 mm diameter field size. In addition, the following planning parameters were used: prescription dose of 45 CGE (Cobalt‐Gray‐Equivalent), distal/proximal margin of 0.7 cm from PTV, aperture margin of 1.0 cm, smearing margin of 1.0 cm, and border smoothing margin of 1.0 cm. These margins are set based on assuming that patients follow strict protocol maintaining constant rectum volume by voiding and filling with 100 cc of saline everyday. In addition, with cone‐beam CT (CBCT)‐based daily image guidance, the interfractional setup and organ motion can be generally limited to 0.4 cm and 0.8 cm, respectively.^(^ ^25^ ^,^ ^26^ ^)^ There is also about 2% range uncertainty due to CT numbers, which in our case adds up to 25cm×0.02=0.5cm. Each uncertainty is added in quadrature^(^ ^17^ ^)^ to determine the appropriate margins.

### E. Analysis

DVHs of PTV, PBM, bowel, rectum and bladder volumes were included in our analysis. Student's t‐test was used for analysis of the different volumes and doses between the IMRT and 3DCPT plans. A two‐tailed *p* value of <0.05 was considered statistically significant. For PTV coverage, the dose conformity index (DCI=1/V95) and dose homogeneity index (DCI=maxdose/prescriptiondose) were calculated as well.

## III. RESULTS

### A. PTV coverage

**Figure 2:**
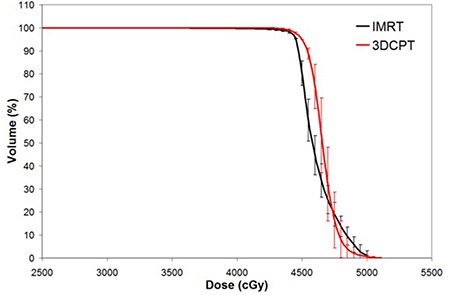
Average planning target volume dose‐volume histogram for intensity‐modulated radiation therapy versus 3D conformal proton therapy techniques. Error bars are one‐standard‐deviation at each corresponding dose‐volume point.

PTV dosimetric conformity and homogeneity were consistently superior with the 3DCPT plans compared with the corresponding IMRT plans. As seen in Fig. 2, the average PTV DVH of 3DCPT falls sharply past the prescription dose, and results in more homogeneous dose, whereas the IMRT DVH has a longer tail past about 47 Gy. In terms of DCI and DHI, 1.00±0.001,1.01±0.02, and 1.10±0.02,1.13±0.01 were obtained for 3DCPT and IMRT, respectively. It is to be noted here that, for IMRT, the DHI obtained are comparable to the similar work reported by Mell et al.,^(^ ^14^ ^)^ where they reported 1.12±0.02. In terms of hot spots, the average difference in V48 (107% of the prescription dose) between the two techniques was 12.6%(p=0.027) higher for the IMRT plans. This observation was consistent across the intact and post‐op cervix groups, as can be inferred from the error bars.

### B. Pelvic bone marrow sparing

**Figure 3:**
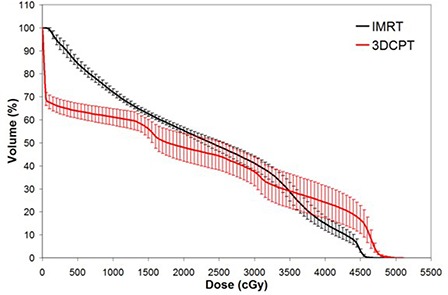
Average pelvic bone marrow dose‐volume histogram for intensity‐modulated radiation therapy versus 3D conformal proton therapy techniques. Error bars are one‐standard‐deviation at each corresponding dose‐volume point.

The 3DCPT plans demonstrated superior ability in sparing PBM in the lower dose range (i.e.,<V30). Figure 3 illustrates the average PBM DVH comparison between the two treatment modalities. As can be seen, the dosimetric differences are the greatest at lower doses and, as the dose increases, IMRT is able to better spare at higher doses. Table 2 summarizes the DVH differences, and the statistical significance, between the two treatment modalities. Of note in the Table is the fact that there are significant differences at V10 and V20 (p=0.001 and 0.04, respectively), as recent studies suggested significant association of acute hematologic events with V10 and V20 for patients undergoing concurrent chemotherapy and IMRT.^(^ ^15^ ^,^ ^16^ ^)^

**Table 2:** Dose‐volume histogram (DVH) differences between intensity‐modulated radiation therapy (IMRT) and 3D conformal proton therapy (3DCPT) for all critical structures evaluated, averaged over the six patient cases (average±standarddeviation). Statistical p values are listed for each corresponding dose‐volume histogram parameters.

	*PBM*		*Bowel*		*Rectum*		*Bladder*	
*DVH*	*IMRT*	*3DCPT*	*IMRT*	*3DCPT*	*IMRT*	*3DCPT*	*IMRT*	*3DCPT*
V10	72.02±1.27	61.19±3.60	72.00±17.80	52.42±13.56	97.77±4.15	98.97±2.51	99.97±0.09	100±0.01
V20	55.44±1.29	48.00±6.01	54.85±13.44	32.88±12.03	94.97±6.47	98.20±3.97	99.56±0.69	80.65±18.15
V30	41.56±3.02	37.47±6.24	41.13±9.31	26.94±10.59	90.39±7.02	91.37±9.24	94.85±8.63	73.72±20.79
V40	15.74±3.04	24.19±7.08	18.54±7.31	21.09±9.31	70.08±7.07	80.02±11.64	62.03±22.78	65.78±21.76
V45	6.54±1.86	16.60±6.11	10.74±4.71	15.67±7.53	48.03±12.09	59.19±6.35	45.13±21.71	56.09±20.65
*p* value								
V10	0.0010		0.0104		0.1808		0.4264	
V20	0.0401		0.0033		0.1082		0.0525	
V30	0.2008		0.0420		0.7831		0.0673	
V40	0.0450		0.2733		0.1012		0.6007	
V45	0.0079		0.0478		0.0605		0.1170	

### C. Bowel, bladder and rectum sparing

**Figure 4:**
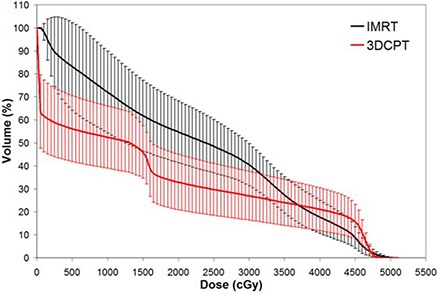
Average bowel dose‐volume histogram for intensity‐modulated radiation therapy versus 3D conformal proton therapy techniques. Error bars are one‐standard‐deviation at each corresponding dose‐volume point.

**Figure 5:**
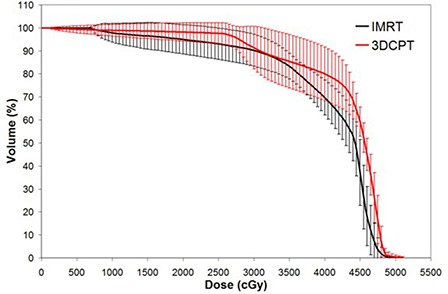
Average rectal dose‐volume histogram for intensity‐modulated radiation therapy versus 3D conformal proton therapy techniques. Error bars are one‐standard‐deviation at each corresponding dose‐volume point.

**Figure 6:**
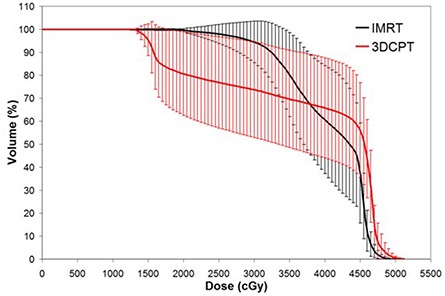
Average bladder dose‐volume histogram for intensity‐modulated radiation therapy versus 3D conformal proton therapy techniques. Error bars are one‐standard‐deviation at each corresponding dose‐volume point.

Figures 4 to 6 show the average DVH of bowel, rectum and bladder, respectively. For bowel and bladder, the pattern of differences is similar to the PBM DVH in Fig. 3, where 3DCPT is superior at organ sparing at lower doses and IMRT is superior at higher doses. This is quantified at various dose levels in Table 2, also. For the rectum, however, IMRT demonstrated superior sparing at all dose ranges, as can be seen in Fig. 5. These observations were consistent across the intact and post‐op cervix groups.

## IV. DISCUSSION

The purpose of this study was to compare IMRT with 3DCPT for low‐dose sparing of PBM, which is an important determinant of acute hematologic toxicity during concurrent chemotherapy and RT treatments.^(^ ^7^ ^–^ ^15^ ^,^ ^16^ ^)^ It is found in this study that forward‐planned conformal proton beams with three gantry angles can be superior in PBM sparing compared with eight‐field inverse‐optimized IMRT plans, given the similar level of PTV coverage. This is no surprise as there are clear‐cut dosimetric advantages to protons as compared with photon beams, such as low entrance dose, spread‐out Bragg peak (SOBP), and finite range of the particles.^(^ ^18^ ^–^ ^22^ ^,^ ^24^ ^,^ ^27^ ^)^ In addition, use of many beam angles for IMRT to spread out the dose distribution inevitably increases the low‐dose spread around the target volume, resulting in spillage of the low doses to the surrounding critical structures. All of these factors contributed to the superiority of 3DCPT plans' sparing of PBM compared with the IMRT plans.

It was also found in this study that IMRT consistently did better in sparing all critical structures at higher doses, including the PBM. At a glance, this may seem an advantage for IMRT but, in fact, this is the direct result of the relatively inferior target coverage demonstrated by the IMRT technique. Recall that Fig. 2 showed PTV DVH comparisons between the two modalities. In this Figure, IMRT demonstrated inferior target uniformity and slower dose gradient past the prescription dose. For IMRT, the hot spots mainly occurred deep in the target volume, whereas the cold spots occurred around the periphery of the PTV. This means that the dose distribution around the edges of the target volume is lower and there is a relatively lower gradient with movement away from the PTV for the IMRT plans. This leads to two consequences: 1) the immediately abutting critical structures receive less high dose irradiation, and 2) OARs receive more low‐dose irradiation farther from the PTV. This is generally what was observed in this study for most of the critical structures (i.e., PBM, bowel and bladder). Therefore, the seeming advantage of IMRT for sparing of high doses to the abutting OARs is due to its relative inferiority in uniform target coverage and sharp dose gradients around its target volume.

For the rectum, however, IMRT was better in sparing at all dose ranges. This was primarily due to the geometric placement of the proton beams (i.e., two posterior oblique beams) that went through most of the rectum volume to reach the PTV. The two posterior oblique beams were strategically placed to minimize the PBM dose and, hence, a tradeoff was made at the planning stage.

To further reduce the dose to the PBM, conventional approaches may be required, such as reducing the CTV‐PTV margin. However, it is unpractical and unsafe to use smaller margins due to the potentially significant inter‐ and intrafractional organ motions observed in the cervix region,^(^ ^25^ ^,^ ^26^ ^)^ and the machine, CT and range uncertainties discussed earlier. A potentially more promising method to reduce the PBM dose, therefore, may be to use more sophisticated proton therapy such as intensity‐modulated proton therapy (IMPT),^(^ ^18^ ^–^ ^21^ ^,^ ^22^ ^)^ although planning and delivery complexities would increase. This technique, which is analogous to IMRT in photon therapy, utilizes spot scanning technology (among others) to optimize each pencil beam by simultaneous optimization of all Bragg peaks from all fields.^(^ ^18^ ^)^ In IMPT optimization, both the beam intensity and energy are varied, as opposed to intensity alone in the IMRT. A series of studies using IMPT by Georg et al.^(^ ^21^ ^,^ ^22^ ^)^ have demonstrated the potential benefits of using IMPT as initial or boost plans for cervical cancer treatment, although they have not investigated PBM sparing explicitly. At the time of planning for this study, we did not have this technology, but as this technology becomes available, we plan to examine the usefulness of IMPT with explicit inclusion of PBM dosimetric constraints during IMPT optimization.

In this study, we defined the pelvic bone marrow as the entire pelvic bones in the treatment region. However, hematopoietically active bone marrow may or may not be distributed evenly across this region for each patient.^(^ ^28^ ^)^ Therefore, identifying and contouring the regions of active bone marrow as an input into IMRT or IMPT optimization may allow identification of better and more robust plans.^(^ ^14^ ^)^ Use of magnetic resonance imaging (MRI), single photon emission CT (SPECT), or other functional imaging technologies could be used for this purpose.^(^ ^29^ ^,^ ^30^ ^)^

Cervical cancer is known to regress over the RT treatment course with up to 79% reduction reported.^(^ ^13^ ^,^ ^31^ ^)^ With the concept of image‐guided adaptive radiation therapy (IG‐ART) being increasingly investigated and accepted,^(^ ^32^ ^,^ ^33^ ^)^ there may be an opportunity to further reduce the PBM dose by regular replanning based on reduced tumor volume during the course of RT. With the combined use of geographical and functional imaging on a regular basis, one may be able to effectively adapt to changing tumor volume, as well as the changing active bone marrow volume.

## V. CONCLUSIONS

The goal of this study was to explore techniques that particularly minimized the low doses in PBM, in light of the recent findings that showed association of V10 and V20 with acute hematologic toxicity in patients undergoing concurrent chemotherapy.^(^ ^15^ ^,^ ^16^ ^)^ Due to its physical characteristics such as low entrance dose, spread‐out Bragg peak and finite particle range of protons, 3DCPT illustrated superior target coverage uniformity and sparing of the low doses in PBM and other organs. However, IMRT showed favorable sparing of all critical structures at higher doses. Therefore, further studies are warranted to exploit the usefulness of protons for other planning/treatment objectives.
